# Evaluation of metabolic syndrome in adults of Talca city, Chile

**DOI:** 10.1186/1475-2891-7-14

**Published:** 2008-05-15

**Authors:** Veronica Mujica, Elba Leiva, Gloria Icaza, Nora Diaz, Miguel Arredondo, Rodrigo Moore-Carrasco, Roxana Orrego, Marcela Vásquez, Ivan Palomo

**Affiliations:** 1 Diabetes and Cardiovascular Program, Maule Health Service, Talca, Chile; 2 Department of Clinical Biochemistry and Immunohematology, Health Sciences School, Universidad de Talca, Talca, Chile; 3 Institute of Mathematics and Physics, Universidad de Talca, Talca, Chile; 4 Institute of Nutrition and Food Technology, Universidad de Chile, Santiago, Chile

## Abstract

**Objective-:**

Insulin resistance (IR) is an important risk factor for type 2 Diabetes Mellitus (DM2) and cardiovascular disease (CVD). Metabolic Syndrome (MS) is a clustering of metabolic alterations associated to IR; however, there is no international consensus for defining its diagnosis. Our objective was to evaluate the prevalence and characteristics of MS identified by the ATP III and IDF criteria in adults from Talca city.

**Research and methods-:**

We studied 1007 individuals, aged 18–74, and residents from Talca. MS subjects were defined according to ATP III (three altered factors) and IDF criteria (patients with waist circumference >80/90 cm (W/M) and two others altered factors).

**Results-:**

The prevalence of metabolic syndrome according to the IDF and ATP III criteria was 36.4% and 29.5%, respectively after adjustment for age and sex. The agreement for both criteria was 89%. The prevalence in men was higher than in women for both MS definitions, although not significant. MS probability increased with age, and the highest risk was in the 57–68 age group (ATP-MS) and 53–72 age group (IDF-MS). Hypertension, high triglycerides and abdominal obesity are the most frequent alterations in MS.

**Conclusion-:**

MS prevalence in adults was higher when diagnosed with IDF than with ATP criterion; in both, age is directly related with the MS presence. The MS subjects showed higher levels of blood pressure, waist circumference and plasma triglycerides. Considering our results, it is worrisome that one third of our population has a high risk of developing DM2 and CVD in the future.

## Introduction

Insulin resistance (IR) is an important risk factor for type 2 Diabetes Mellitus (DM2) and cardiovascular disease (CVD) [1-3]. It is characterized by a decrease in biologic insulin action, so plasmatic insulin levels are higher to maintain normal glucose plasma levels [4]. Several studies support the relationship between insulin resistance (IR) and CVD [5-8]. However, insulin sensitivity measurement is complex and expensive, so the model proposed by Mathews et al. [9] that estimates the degree of IR at baseline by the homeostasis model assessment (HOMA-IR) has acquired importance. Metabolic Syndrome (MS) is a clustering of metabolic alterations associated to IR, but conceptual differences exist between the currently available definitions [10,11]. Reaven [2] was the first to describe this combination as a syndrome that he called IR syndrome or simply "X Syndrome"; later the World Health Organization (WHO) named it Metabolic Syndrome. This definition based the diagnosis of MS on the presence of hypertension, hypertriglyceridemia, low HDL cholesterol (HDLc), hyperglycemia and/or, hyperinsulinism, but also added the waist/hip ratio and urinary albumin excretion as components of this syndrome [12,13].

The progressive increase in obesity, CVD and MS prevalence motivated the National Cholesterol Education Program (NCEP) on its third panel: *Treatment of High Blood Cholesterol in Adults *(ATP III) [14], to propose clinical criteria to define MS by the presence of at least three altered factors: High blood pressure (BP), hypertriglyceridemia, low HDLc, high plasmatic glucose and abdominal obesity. This definition was simple, so various prospective studies adopted the definition and determined its relation to CVD, but later the NCEP criteria were criticized because the identification of those affected is strongly influenced by ethnicity [15-17]. The thresholds were selected based on evidence from studies in Caucasian populations and variability among ethnic groups was not taken into account since waist circumference and body composition are different in Asian and Hispanic populations [18-20].

The International Diabetes Federation (IDF) proposed a new definition for MS [21] that is based on the importance of abdominal obesity as a condition that must be present in all subjects with the syndrome, and that the threshold for waist circumferences must be defined in each country for its own ethnic groups [22]. In Latin-American populations, since there are no epidemiologic studies to define the best waist circumference cut-off points, it was suggested to be considering the same values as those of south-Asiatic populations who consider altered waists being over 80 cm in women and 90 cm in men. Consequently IDF-MS is defined as abdominal obesity with the presence of two altered factors, as is ATP-MS. IDF considered abnormal glucose over 100 mg/dl as was suggested by the American Diabetes Association (ADA) [23]. Furthermore, the American Association of Clinical Endocrinologist (AACE) and the American Heart Association (AHA) agree on the lower glucose level but have decided to keep the ATP III diagnosis criteria [24] for MS.

Although there is controversy about the diagnosis, most of the authors agree that the presence of MS is associated with a higher risk of developing DM2 and CVD [25-27] and they include as important factors for the diagnosis, the alterations in waist circumference, BP, HDLc, triglycerides and glucose levels.

Due to the high incidence of MS in Chile and in Talca [28], we decided to study the prevalence and characteristics of MS using the IDF and ATP diagnosis criteria and the relative importance of their individual components.

## Research design and methods

We studied 1007 subjects, aged 18 to 74 years old, residents of Talca, Chile [29]. The study group was selected from a probabilistic polietapic sampling scheme. In the first step, blocks were numbered according census district, then with a simple randomization 361 blocks were selected in the city. In the second step, by a systematic selection procedure, 8 houses per block were selected and a trained surveyor selected one subject per house by using the Kish table [30]. Anthropometric and arterial blood pressure measurements and blood extractions (for glucose and lipids) were performed at the Clinical Laboratory of the Health Sciences School at the Universidad de Talca. Informed consent was signed by all participant subjects. The protocol was approved by the ethic committee from Universidad de Talca and Health Service of Maule, Chile.

The diagnosis criteria used for MS were: **a) According to ATP definition (ATP-MS)**: three or more of the following factors: 1) Blood pressure: Systolic ≥ 130 mmHg and/or diastolic ≥ 85 mmHg and/or subjects who received anti hypertension drug therapy. 2) Triglycerides: ≥ 150 mg/dl; 3) HDL cholesterol: <40 in men and <50 mg/dl in women; 4) Plasmatic glucose: ≥ 100 mg/dl, and/or subjects who received anti diabetic drug therapy and 5) Waist circumference: >102 cm in men and >88 cm in women; and **b) MS according to IDF definition (IDF-MS)**: Waist circumference over 90 cm in men and over 80 cm in women, and the presence of any two altered factors described above for the ATP criteria.

### Statistical analysis

MS prevalence was adjusted according to population distribution by age and sex according to the 2002 national census. Means differences between sexes were evaluated using the Student t-test. Agreement analysis was performed to compare diagnosis criteria with the Mc-Nemar test and the Kappa statistic test [30]. We adjusted a generalized additive model to evaluate the probability of MS associated with age under both criteria [31]. We used SAS 9.1.3, software for statistical analysis.

## Results

From the 1007 total subjects, 37.5% presented overweight,32.6% obesity, and 41.1% abdominal obesity; 37% had high BP and 26.3% impaired fasting glucose; and 40.1% had high triglycerides and 21.5% low HDLc. The anthropometric and biochemistry characteristics by sex are shown in Table 1. There was no difference in age and BMI between men and women; however, all the other parameters were significantly different.

**Table 1:** Characteristics of studied subjects by sex (n = 1007).

	**Men**	**Women**
	**Mean ± SD**	**Mean ± SD**

N	339	668
Age (years)	44.3 ± 15.1	45.6 ± 13.5
BMI (kg/mt^2^)	28.3 ± 4.5	28.5 ± 5.8
Waist circumference (cm)	96.3 ± 12.0	89.2 ± 13.0*
Systolic blood pressure (mmHg)	133.8 ± 19.6	124.5 ± 20.8*
Diastolic blood pressure (mmHg)	80.7 ± 12.4	75.8 ± 11.0*
Triglycerides (mg/dl)	190.4 ± 175.2	147.6 ± 95.4*
HDLc (mg/dl)	45.8 ± 12.3	55.0 ± 15.3*
Glucose (mg/dl)	100.2 ± 29.2	93.7 ± 24.1*

BMI: body mass index; HDLc: HDL cholesterol

*Student t-test: p < 0.05

### Prevalence of metabolic syndrome

**Figure 1:**
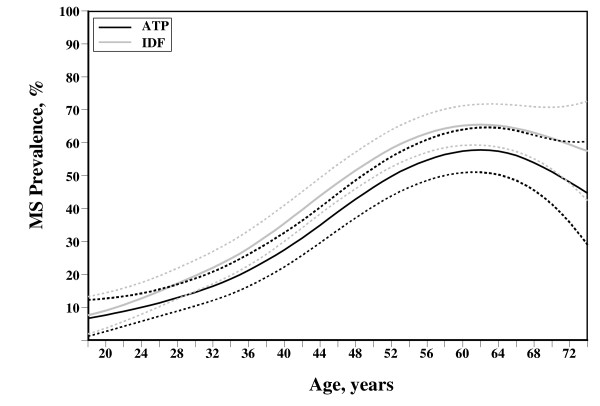
Prevalence of Metabolic Syndrome according ATP and IDF definition by age.

449 (44.6%) subjects presented MS including the ATP and/or IDF criteria and 75% of them presented MS according to both criteria. The average age in these patients was 51.6 ± 11.8 years for patients diagnosed with ATP criteria and 51.1 ± 12.0 years for individuals diagnosed with IDF. The IDF criteria detected more prevalence of MS than the ATP criteria (Table 2). 357 subjects (35.5%) were diagnosed as having MS by ATP criteria and 430 patients (42.7%) by IDF criteria. After adjusting for age and sex, the prevalence of MS according to IDF was 36.4%, which is significantly higher than the prevalence found by ATP criteria (29.5%) (p = 0.001). Men presented a tendency for higher MS prevalence, however, these differences were not significant (IDF: 39.0% y 34.0%, p = 0.138; ATP: 30.1% y 29.0%, p = 0.786 in men and women, respectively). The MS risk for age according to both criteria is shown in Figure 1. MS probability increased with age, with the highest risk in the 57–68 age group (ATP-MS) and 53–72 age group (IDF-MS).

**Table 2:** Characteristics of subjects with Metabolic Syndrome ATP and IDF definition

	**Male**		**Female**	
	**ATP**	**IDF**	**ATP**	**IDF**

N	117	155	240	275
BMI (kg/m^2^)	31.0 ± 4.3	30.5 ± 3.9	32.5 ± 5.8	31.9 ± 5.6
Waist circumference (cm)	104.5 ± 11.2	103.0 ± 9.9	99.2 ± 11.0	97.7 ± 10.9
Systolic blood pressure (mmHg)	143.7 ± 18.9	141.1 ± 19.3	136.5 ± 21.8	135.3 ± 22.0
Diastolic blood pressure (mmHg)	86.8 ± 12.0	85.9 ± 12.4	81.5 ± 11.1	80.8 ± 11.1
Triglycerides (mg/dl)	282.1 ± 250.9	260.9 ± 227.8	201.3 ± 115.1	201.9 ± 117.2
HDLc (mg/dl)	40.7 ± 11.4	40.4 ± 10.3	46.7 ± 11.6	47.5 ± 12.5
Glucose (mg/dl)	115.6 ± 39.1	111.9 ± 38.3	106.0 ± 33.1	104.5 ± 32.3

BMI: body mass index; HDLc: HDL cholesterol

Data are means ± SD unless other was indicated.

In relation to the distribution of the involved factors, we observed a similar behavior for ATP and IDF MS that is expected if we consider that there is an important overlap among the subjects. If we consider ATP vs. IDF, respectively, high blood pressure (83.8% vs. 77.9%); high triglycerides (74.5% vs. 71.6%) and increased waist circumference (77.9% vs. 100%) were the major components found in subjects with MS, independent of the criteria, followed by hyperglycemia (57.7% vs. 51.4%) and low HDLc, in both men and women (65.6% vs. 61.9%).

### Analysis of the concordance from the different syndrome status

When we compare the concordance between both diagnostic criteria, the Kappa statistic was 0.77 (95% CI 0.73–0.81), which suggests a good concordance between the ATP and the IDF diagnosis for MS. We evaluated the discrepancies in the diagnosis and we observed that the IDF criteria were more likely to diagnose MS as positive as the ATP criteria (McNemar test: p < 0.001); 9.1% was diagnosed with MS using only IDF, and 1.9% was diagnosed with MS using only ATP criteria.

## Discussion

MS presents a high prevalence in the world and in Chile [25]. In Latin America there are no other published studies about MS-IDF with the present waist circumference cut-off points. Park et al. [32] reported a MS-IDF prevalence of 13.5% in men and 15.0% in women in the Korean population, which is remarkably lower than our prevalence. Boehm et al. [33] found in studies that older German people (over 55 years old) showed an important discordance between both diagnosis criteria: in females a prevalence of 24% with ATP and 46% with IDF and in males 28% y 57%, respectively. These differ from our results that found 75% of concordance for both criteria.

The MS prevalence varies depending on the diagnosis criteria; most are higher with IDF than ATP. Also there is marked disagreement if we consider the geographic regions and the ethnic origin, so it would be interesting to compare our findings with similar populations. However, we did find two studies of Hispanic populations that are similar to ours. The Chilean National Health Report showed that MS was present in 27% of the Maule Region population, which is similar to the 31% reported for Americans of Hispanic origin [34]. Both studies used ATP criteria with glycaemia over 110 mg/dl so the criterion was not exactly the same as in our study where we used 100 mg/dl as the cut-off point. In spite of this, those results are similar to the 29% found by our group for ATP-SM.

Other groups have reported important differences in the prevalence of MS by sex. The Americans did not find differences in the NCEP study [34], neither did the Philippines [35], the Spanish in the Canary Islands [36] or the Koreans [32]. We did not find significant difference by sex, even though with IDF criteria women present a tendency to show a higher prevalence. Nevertheless, there are many populations where there are marked differences by sex, as for the Iranians in the Teheran study; where a prevalence of 42% in women and 24% in men was found [37]. Similarly, the Indian study reported 46.5% in women and 36.4% in men [38]. A Lithuania study, using IDF criteria, found a prevalence of 28.1% in men and 16.6% in women [39], similar to the San Antonio Heart Study that had 28.9% in men, and 20.8% in women [6]. Also, a similar prevalence was reported by Magi in Italians [40], but they found greater ATP-MS in women (27%) than in men (22%).

The most frequent individual factor is high blood pressure in most of the populations studied; for instance, Americans [8], Lithuanians [40], Koreans [32] and Philippines [35], and also in our MS subjects for both sexes. Table 3 shows a comparison of prevalence of MS according ATP criteria in different areas. Talca results show a higher prevalence compared to that found in other Chilean studies and in other countries. Bustos et al., [41] showed 10.1% of MS prevalence in a semirural population of Chile (Limache) and Brasil (Riberao Preto), 7.6%. These populations had an average age of 24 years and the prevalence found is similar to the data presented in Figure 1, which shows results by age.

**Table 3:** Comparative prevalence (%) of metabolic syndrome

	Men	Women
Talca	29.5 ± 2.5	28.2 ± 1.7
Chile	23.0 ± 1.3	22.3 ± 1.2
*USA	24.0 ± 0.7	23.4 ± 0.6
Norway	26.8 ± 0.6	25.0 ± 0.6
*Korea	24.6 ± 1.0	28.1 ± 0.9

Data are presented in prevalence (S.E)

*Plasma fasting glucose level ≥ 110.0 mg/dL

Finally, we believe that the evidence remains controversial concerning the definition of MS and its relationship to CVD [42,43]. Consequently, finding the best diagnostic criteria and their significance require more study. On other hand, the healthy waist circumference cut-off point is a problem for different ethnic groups and populations. In this article, we used the IDF-MS classification for waist circumference based on the limit proposed for Latin America, which was homologated from the Asian population. However, we believe that the Chilean physical constitution is more similar to the European than Asian population. Thus, it is necessary to establish our own tables based on local population studies.

## Competing interests

The authors declare that they have no competing interests.

## Authors' contributions

VM study design, sample selection and attending physician, data collection, analysis and interpretation and writing of the manuscript; EL study design, laboratory analysis coordination, data collection, analysis and interpretation and writing of the manuscript; GI statistical sample design and analysis; ND statistical analysis; MA results analysis, discussion and writing of the manuscript; RM laboratory analysis; RO patients management in field site and collection and storage of samples; MV management of the field site and IP patient care and analysis and interpretation and writing of the manuscript. All the authors review the article and approve the final manuscript.
